# Characterization of the Biomechanical Properties of Skin Using Vibrational Optical Coherence Tomography: Do Changes in the Biomechanical Properties of Skin Stroma Reflect Structural Changes in the Extracellular Matrix of Cancerous Lesions?

**DOI:** 10.3390/biom11111712

**Published:** 2021-11-17

**Authors:** Frederick H. Silver, Nikita Kelkar, Tanmay Deshmukh, Kelly Ritter, Nicole Ryan, Hari Nadiminti

**Affiliations:** 1 Department of Pathology and Laboratory Medicine, Robert Wood Johnson Medical School, Rutgers, The State University of New Jersey, Piscataway, NJ 08854, USA; 2 OptoVibronex, LLC., Allentown, PA 18104, USA; nuk1@scarletmail.rutgers.edu (N.K.); tmd24895@gmail.com (T.D.); 3 Dermatology, Summit Health, Berkeley Heights, NJ 07922, USA; KRitter@summithealth.com (K.R.); Nryan@summithealth.com (N.R.); HNadiminti@summithealth.com (H.N.)

**Keywords:** collagen, fibrous tissue, stroma, actinic keratosis, basal cell carcinoma, squamous cell carcinoma, epithelial–mesenchyme transition, vascular mimicry, epithelial cells, extracellular matrix

## Abstract

Early detection of skin cancer is of critical importance since the five-year survival rate for early detected skin malignancies is 99% but drops to 27% for cancer that has spread to distant lymph nodes and other organs. Over 2.5 million benign skin biopsies (55% of the total) are performed each year in the US at an alarming cost of USD ~2.5 B. Therefore there is an unmet need for novel non-invasive diagnostic approaches to better differentiate between cancerous and non-cancerous lesions, especially in cases when there is a legitimate doubt that a biopsy may be required. The purpose of this study is to determine whether the differences in the extracellular matrices among normal skin, actinic keratosis (AK), basal cell carcinoma (BCC) and squamous cell carcinoma (SCC) can be assessed non-invasively using vibrational optical coherence tomography (VOCT). VOCT is a new diagnostic technology that uses infrared light and audible sound applied transversely to tissue to measure the resonant frequencies and elastic moduli of cells, dermal collagen, blood vessels and fibrous tissue in skin and lesion stroma without physically touching the skin. Our results indicate that the cellular, vascular and fibrotic resonant frequency peaks are altered in AK, BCC and SCC compared to those peaks observed in normal skin and can serve as physical biomarkers defining the differences between benign and cancerous skin lesions. The resonant frequency is increased from a value of 50 Hz in normal skin to a value of about 80 Hz in pre- and cancerous lesions. A new vascular peak is seen at 130 Hz in cancerous lesions that may reflect the formation of new tumor blood vessels. The peak at 260 Hz is similar to that seen in the skin of a subject with Scleroderma and skin wounds that have healed. The peak at 260 Hz appears to be associated with the deposition of large amounts of stiff fibrous collagen in the stroma surrounding cancerous lesions. Based on the results of this pilot study, VOCT can be used to non-invasively identify physical biomarkers that can help differentiate between benign and cancerous skin lesions. The appearance of new stiff cellular, fragile new vessels, and stiff fibrous material based on resonant frequency peaks and changes in the extracellular matrix can be used as a fingerprint of pre- and cancerous skin lesions.

## 1. Introduction

Approximately 5 million patients develop skin cancer in the United States each year [1] and current predictions are that by age 70, one out of five Americans will develop some form of skin cancer [1,2,3,4]. Early detection is of critical importance since the five-year survival rate for early detected skin malignancies is 99% but drops to 27% for cancer that has spread to distant lymph nodes and other organs [5,6,7,8]. Skin lesion analysis is performed by specialized dermatologists through visual or dermoscopic evaluation [9,10,11]. However, these techniques have a poor diagnostic ability and over 2.5 million benign skin biopsies (55% of the total) are performed each year at an alarming cost of USD ~2.5 B [12,13]. In addition, lesion margins are often miscalculated, and 5–20 mm of extra healthy skin is normally removed during excision leading to substantial cosmetic impairment [14]. Therefore it is important to elucidate whether changes in the biophysical properties of extracellular matrix (ECM) surrounding epithelial lesions is a physical biomarker or fingerprint that can be used to characterize different lesion types.

Basal cell carcinoma (BCC) is the most prevalent form of skin cancer worldwide. In Europe, BCC incidence has been increasing by approximately 5% annually over recent decades [15] It is a major burden on healthcare systems [16,17] and is most commonly seen in the head and neck of skin cancer patients [15,18]. BCC is a cutaneous malignant proliferation that has been postulated to derive from the basal cell layer and outer root sheath of the hair follicle. It accounts for 90% of all skin cancers in the United States [2]. In addition to BCC contributing to about 4 million biopsies a year, actinic keratosis (AK) is a major pre-cancerous lesion that is often biopsied.

AKs are categorized as pre-neoplastic lesions and are considered as having characteristics of malignancy since their genesis is similar to that observed in spinocellular carcinoma, including loss of polarity, nuclear pleomorphism, dysregulated maturation, and increased number of mitoses [18]. AKs are considered a part of the evolutionary spectrum of SCC. The prevalence of actinic keratoses ranges from 40% to 60% in Australia among Caucasians over 40 years of age, and 11.5% to 26% in the United States in individuals over 30 years of age [19].

SCC accounts for the most prevalent form of non-melanoma metastatic skin cancer; therefore, recognition and treatment of early SCC is important for the prevention of neoplastic progression. Although there is some overlap between dermoscopic features of AK, in situ SCC, and microinvasive SCC, there are also some important indicators that can assist in the diagnosis of SCC and its subsequent management [19].

The current landscape of skin cancer diagnostic devices is dominated by optical coherence tomography (OCT) that provides tissue morphology imagery at a higher resolution than modalities such as magnetic resonance imaging or ultrasound, but used alone cannot differentiate tissues components and cannot provide quantitative data on tissue stiffness [20,21]. Other imaging techniques such as confocal microscopy and histopathology give high-resolution images but do not provide any quantitative cellular and collagen mechanical data, and are destructive techniques (specifically histopathology) [22,23]. Emerging techniques include magnetic resonance elastography (MRE) [24], optical coherence elastography (OCE) [25,26,27,28,29] and ultrasound elastography (UE) [30,31]. All of these techniques are non-invasive and provide high-resolution skin imaging; however, they are not readily available in imaging and dermatology departments and they present limitations in the accuracy of stiffness measurements. Most of these techniques calculate a shear stiffness rather than tensile properties of the skin and are all based on the false assumption that the tissue is a linear elastic solid and that Poisson’s ratio is 0.5 (stretching occurs at constant volume), resulting in calculation errors and uncalibrated shear stiffness measurements. In addition, they can only penetrate to the level of the dermis and not to deeper structures [23,24,25,26,27,28,29,30]. VOCT in combination with ultrasound was shown to provide biophysical markers on tissues as deep as 8 cm [32,33].

The purpose of this study is to determine whether the differences in the biophysical properties of ECM in normal skin, AK, BCC and SCC can be discerned non-invasively using vibrational optical coherence tomography (VOCT). VOCT is a new diagnostic technology that uses infrared light and audible sound applied transversely to the skin surface to measure the resonant frequencies and elastic moduli of cells, dermal collagen, blood vessels and fibrous tissue in skin and skin lesions without physically touching the tissue [33,34,35,36,37].

## 2. Methods

### 2.1. Subjects

**Figure 1:**
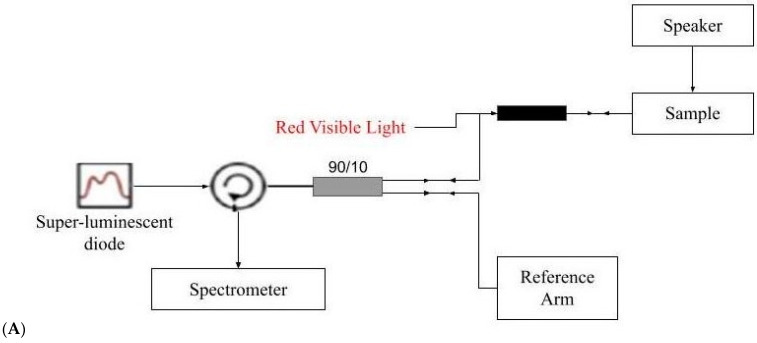

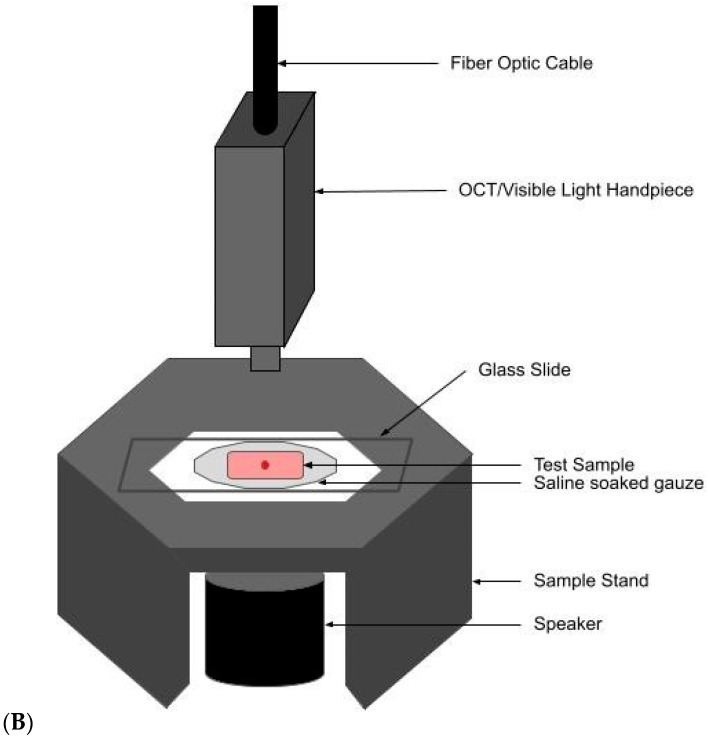
(**A**) Block diagram of the setup of an OCT device modified to do vibrational optical coherence tomography (VOCT). The speaker shown, which is 2 inches in diameter, provides audible sound through a computer-driven app to vibrate the sample between 30 and 300 Hz. The displacement of the sample at each frequency is obtained from amplitude data collected and stored from raw images created by the OCT. (**B**) Schematic drawing of the specimen holder and the OCT handpiece used to collect the VOCT data at each frequency on skin lesion biopsies. The biopsy sample is placed on saline-wetted gauze to prevent dehydration during testing.

Suspicious skin lesions identified by dermoscopy in the Dermatology Clinic at Summit Medical Group (Berkeley Heights, NJ) were biopsied and studied in vitro using VOCT after informed consent was obtained. Normal skin on the outside edges of biopsies with clear margins was studied using VOCT and the vibrational OptoScope (OptoVibronex LLC., Allentown, PA, USA) as the control. Biopsies examined included skin from the hands, arms, neck and legs. The resonant frequencies of the components of skin were measured in vivo by mounting the OCT handpiece (Lumedica, Inc. Durham, NC, USA) on a custom-built stage on which the skin biopsy rested (Figure 1). The subjects studied ranged in age from 45 to 98 years old.

During dermoscopy these lesions appeared to contain piles of cells, superficial blood vessels or abnormal color. They were obtained from the arms, legs, abdomen and necks of subjects seen in Dermatology at Summit Health. The biopsied lesions were studied blindly by VOCT without identification of the age and sex of the patient. In total over 100 lesions were studied as part of an IRB-approved study and only lesions with a diagnosis of AK, BCC and SCC (84 total lesions) were analyzed in this study. Thirty-three biopsies of complete excisions and fifty-one Mohs sections were examined using VOCT. All subjects signed consent forms prior to enrolling in the study. VOCT measurements were made on an area of about 0.0625 mm^2^ as discussed below. The samples were studied by VOCT within 5 min of harvesting by the Dermatologist and kept wet using moist saline impregnated gauze during testing. The biopsies were placed on a custom built stage (OptoVibronex, LLC.) with the speaker placed beneath the stage as shown in Figure 1.

Once VOCT studies were conducted, the sample was immersed in fixative and transported to the pathology lab for diagnosis. Histopathology on skin biopsies was conducted by a dermatopathologist after routine dehydration in alcoholic solutions, embedding in paraffin, thin sectioning and staining with H&E (Fischer Scientific, Waltham, MA, USA). Mohs thin sections were processed after fixation by frozen sectioning and H&E staining. They were reviewed by a trained Mohs dermatopathologist who conducted the pathological analysis.

### 2.2. OCT Images and Scans of Pixel Intensity Versus Depth Measurements

OCT image collection was accomplished using a Lumedica Spectral Domain OQ 2.0 Labscope (Lumedica Inc., Durham, NC, USA) operating in the scanning mode at a wavelength of 840 nm. The device generates a 512 × 512 pixel image with a transverse resolution of 15 micrometers and an A-scan rate of 13,000/s. All gray scale OCT images were color-coded to enhance the image details. For the pixel intensity versus depth plot, the surface of the sample was traced and the average of pixel values was calculated along the surface of the sample, which was then plotted against the depth. For curved biopsy specimens, the tracing was carried out parallel to the surface of the image.

The pixel intensities obtained from the gray scale image were plotted versus depth for each sample studied. The enhanced OCT images used darker colored (blue and purple) regions to reflect lower pixel intensities while the lighter (yellowish) regions reflected higher pixel intensity regions. Pixel intensities were processed using image J software (NIH, Bethesda, MD, USA), analyzed with a MATLAB program (MathWorks, Inc, Natick, MA, USA), and plotted versus skin depth. Previous studies have shown that the images of normal skin and cancerous lesions seen by OCT correlate with the histological images seen in sections cut from tissue biopsies [20,21].

### 2.3. Measurement of Resonant Frequency and the Elastic Modulus

The OQ Labscope (Lumedica, Inc.) was modified as shown in Figure 1 by adding a 2-inch diameter speaker (J.Y.M. Electronics Co., Shenzhen, China) to vibrate the sample in the VOCT studies. The Labscope was also modified to collect and store single raw image data that were used to calculate sample displacements (amplitude information) from A-line data. The data were processed using MATLAB software as discussed previously [33,34,35,36,37]. The displacement of the tissue is detected by measuring the frequency dependence of the deformation based on the reflected infrared light. The result is a spectrum of displacements for specific tissue components as a function of frequency of the applied sound; the resonant frequency of each tissue component was assigned previously [33,34,35,36,37].

For VOCT measurements, the raw images were captured and were imported into MATLAB and by applying signal processing filters the elastic vibrations for each frequency were isolated to calculate the amplitudes. These amplitudes were plotted against the frequency of vibrations.

The resonant frequency of a tissue component is defined as the frequency at which the maximum displacement is observed in the amplitude data. The measured resonant frequencies are converted into elastic modulus values using a calibration equation (Equation (1)) developed based on in vitro uniaxial mechanical tensile testing and VOCT measurements made on the same tissue as reported previously [33,34,35,36,37]. The resonant frequency of each sample is determined by measuring the displacement of the tissue resulting from sinusoidal audible sound driving frequencies ranging from 30 Hz to 300 Hz, in steps of 10 Hz. The peak frequency (the resonant frequency), *fn*, is defined as the frequency at which the displacement is maximized.

(1) $$\mathrm{Soft}\;\mathrm{Tissues}\;E*d=0.0651*\left(fn^{2}\right)+233.16$$

Calibration studies using in vitro uniaxial tensile testing and VOCT measurements were used to develop Equation (1) for soft tissues. Since most soft tissues have a density very close to 1.0, Equation (1) is valid for the majority of tissues found in the body; where the thickness d is in m and is determined from OCT images, $fn^{2}$ is the square of the resonant frequency, and E is the elastic modulus in MPa as discussed previously [33,34,35,36,37].

## 3. Results

This study was initiated to determine if measurements of the resonant frequencies of cellular (50 Hz), dermal collagen (100 Hz), vasculature (150 Hz) and fibrous tissue (200–260 Hz) could be used to differentiate between normal skin and skin lesions using VOCT. These component resonant frequencies were established based on studying a variety of tissues using VOCT and ultrasound. The diameter of the infrared light beam is about 0.25 mm so that small areas (0.0625 mm^2^) of the lesion can be quantitatively analyzed. Only lesions that could be clearly located based on the visual camera and OCT images were included in the study data to ensure that the measurements reported were collected on well-characterized areas of the lesions. We have shown previously that OCT images of lesions appear as circular to elliptical spots in the skin and that these spots correlate with lesion images seen by histopathology [20,21]. Some Mohs sections were too thin (less than 0.1 mm in thickness) to clearly visualize the lesion based on the OCT images and were not included in the data analysis.

**Figure 2:**
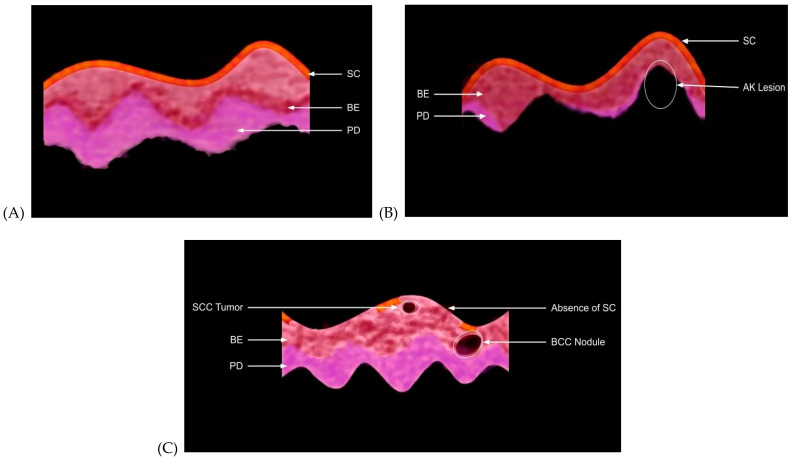
Schematic diagram of OCT color-coded images describing the differences between normal skin (**A**), AK, BCC and SCC (**B**,**C**) derived from previous study results [20,21]. Normal skin (**A**) contains an epidermis composed of a stratum corneum (SC) on top, an epithelial layer that derives from the basal cell layer (BE), rete pegs that undulate at the basal cell–papillary dermal layer interface (PD). The papillary dermis contains dermal collagen with a resonant frequency of 100 Hz. Pre- (AK) (**B**) and cancerous lesions (**C**) (BCC and SCC) contain nodular and linear tumors. Cancerous tumors (BCC and SCC) contain reduced amounts of stratum corneum and lack the undulating rete pegs. The dermal collagen fibers with a resonant frequency of 100 Hz are replaced by lesions with dense fibrous tissue (black spots) that reflect more light back to the detector. The stiffness of the tumor collagen can be determined by measuring the resonant frequency of the material in the black spots. Resonant frequency peaks in cancerous lesions occur at 260 Hz as a result of stiffening of the collagen deposited by tumor cells.

**Figure 3:**
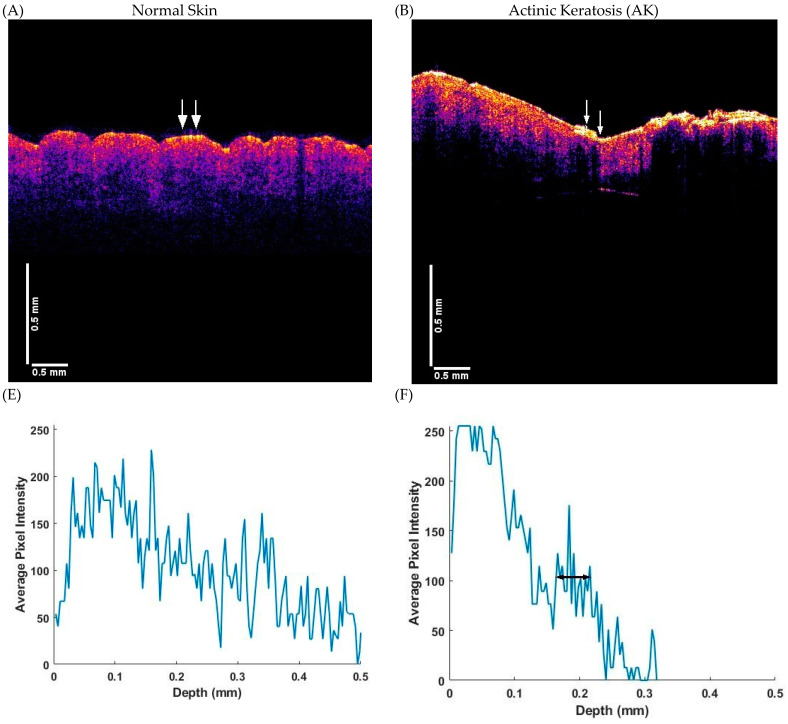

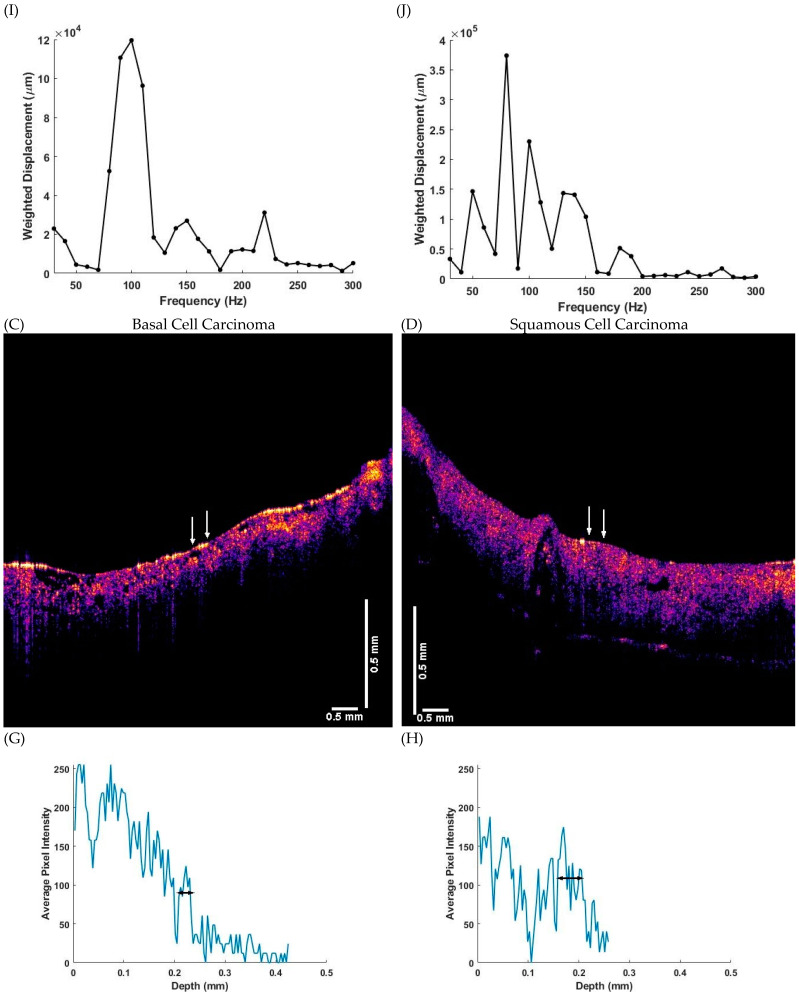

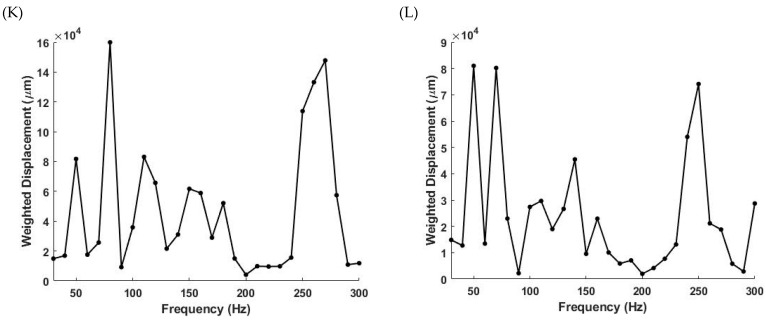
Comparison of the color-coded OCT images (**A**–**D**), pixel intensity versus depth plots (**E**–**H**), and weighted displacement in μm versus frequency plots in Hz (**I**–**L**) for the areas marked by the arrows for normal skin, AK, BCC and SCC obtained using VOCT. The arrows in (**A**) through (**D**) mark the locations at which the measurements were made on biopsies with the results presented in panels (**E**) through (**L**). Note the plateau marked with a horizontal line in (**F**) through (**H**) in the pixel intensity versus depth plots occurs at a depth of about 0.2 mm. This is approximately at the interface between the epidermis and the papillary dermis. In normal skin and AK there is no fibrous tissue peak at 260 Hz (**I**,**J**) while in the cancerous lesions ((**K**), BCC and (**L**), SCC) note the large peak at 260 Hz suggesting that this peak is where stiff fibrous tissue is deposited. The 260 Hz peak is one of the markers that can be used to differentiate between benign and cancerous skin lesions.

Figure 2 shows a schematic diagram of typical OCT images of normal skin and AK, BCC and SCC lesions based on previous publications [20,21]. Note the lesions can be circular (nodular) or they can be elliptical or linear and involve the basal cell layer (BCC) or squamous cells (AK and SCC) or both. Note while the lesions appear as black spots in the OCT image, it is not possible to discern the lesion type based only on the OCT image. Figure 3 shows a combination of typical OCT images for normal skin, AK, BCC and SCC and the exact location (see arrows) where the VOCT data were taken; the pixel intensity as a function of depth and typical plots of weighted displacement versus depth were collected at the points between the arrows. Typical weighted displacement versus frequency plots from these locations is shown for normal skin, AK, BCC and SCC. The peaks found in normal skin were assigned based on previous publications [20,21]. Note in normal skin the cellular peak (50 Hz) is very small or non-existent while the dermal collagen peak (100 Hz) predominates (Figure 3I). The vascular peak (150 Hz) and fibrous collagen peaks (260 Hz) are present but are small unless the measurement is made over a blood vessel or a wound as previously reported [20,21]. The plot of weighted displacement versus frequency for AK (Figure 3J) is very different from that of normal skin, with a peak present at 50 Hz (elastic modulus 1.02 MPa) and a prominent one at 80 Hz (1.91 MPa). The blood vessel peak at 130 Hz (4.26 MPa) is lower than that observed in normal skin and over blood vessels (150 Hz and 5.51 MPa).

The color-coded OCT image of a BCC (Figure 3C) shows a dark spot (arrows) and the plot of pixel intensity versus depth (Figure 3G) shows a plateau at about 0.2 mm. The dark spot occurs at a depth of about 0.2 mm, the location of the epidermal–dermal interface and roughly corresponds to the plateau in the pixel intensity versus depth plot (Figure 3G). The correlation between the dark spots and locations of skin lesions was verified by histopathology for BCC and SCC skin lesions [35,36]. Whether the lesion is benign or cancerous cannot be identified based on the OCT image; however, the resonant frequency and modulus measurements on an area containing the lesion identified in Figure 3J–L provide additional information about the lesion. The peak at 260 Hz for BCC and SCC has a modulus of 15 MPa well above any moduli values seen in normal skin and AK. 

Figure 3 also contains a color-coded OCT image of a typical SCC (Figure 3D) and a plot of pixel intensity versus depth plot for SCC (Figure 3H). Note the arrows pointing out the lesion location in the SCC image (Figure 3D) and the horizontal line to the plateau in the pixel intensity versus depth plot (Figure 3H) where the lesion is observed. Note the location of the lesion and fibrous tissue in AK, BCC and SCC is usually at a depth of about 0.2 mm in both the OCT image and plot of pixel intensity versus depth. The plot of weighted displacement versus frequency for an SCC lesion (Figure 3L) also has resonant frequency peaks at 80, 130 and 260 Hz similar to those seen in BCC (Figure 3K).

Table 1 lists the statistical difference analyses between the 80 Hz peak heights for normal skin, AK, BCC and SCC. The data in Table 1 indicate that the peak heights of AK, BCC and SCC are statistically larger than that of normal skin at a confidence level of 0.965 or greater.

All data in Table 2 through to 6 are based on the same sample sizes as stated in Table 1. Table 2 illustrates that the 260 Hz peak height for AK is statistically smaller at a confidence level greater than 99% compared to the 260 Hz peak heights for BCC and SCC; the BCC 260 Hz peak height is statistically larger than the SCC peak height at a confidence level greater than 98%.

Results listed in Table 3 and Table 4 indicate that normal skin has a lower ratio of the 80 to 100 Hz peaks (Table 3) and the ratio of the 100 and 130 Hz peaks (Table 4) when compared to AK, BCC and SCC at a confidence level of 98% or better. AK has a lower ratio of the 260 and 100 Hz peaks (Table 5) and 150 and 100 Hz peaks (Table 6) at a 94% or greater confidence level compared to BCC and SCC.

These results suggest that AK, BCC, and SCC have larger 80 Hz peaks than normal skin, BCC and SCC have larger 260 Hz peaks than AK, and the 260 Hz peak is statistically higher for BCC than it is for SCC. In addition, the ratio of the 260 to 100 Hz peaks and the ratio of the 130 and 100 Hz peaks are higher for BCC and SCC compared to AK and normal skin. The reported peak heights and ratios can be used to differentiate among normal skin, AK, BCC and SCC.

## 4. Discussion

Cancer cells and cancerous tissue were reported in the literature to be stiffer than normal tissue [38,39,40,41,42]. In this study, we attempt to determine whether this change in stiffness can be measured non-invasively and interpreted in terms of changes in cellular and ECM properties in lesions that are seen in the clinic and normally biopsied. This requires a method to measure cellular and tissue stiffness in vivo and to be able to relate changes in the biomechanical properties of ECM to changes in the amounts and nature of the epithelial stroma.

We reported a new technique to measure the stiffness of the components of ECM non-invasively in vivo using vibrational optical coherence tomography (VOCT) [20,21,32,33,34,35,36,37]. This technique can be used to image and measure the stiffness of cellular, dermal collagen, vascular and fibrous tissue compositions of benign and malignant skin lesions [20,21]. Preliminary results indicate that the cellular peak resonant frequency and stiffness is increased from 50 Hz (1.02 MPa) in normal skin to about 80 Hz (1.91 MPa) in pre- and cancerous lesions. The peak at 200–260 Hz is associated with deposition of varying amounts of fibrous tissue in wounds, BCC and SCC lesions [20,21] and in an animal model of fibrosis and fibrous tissue in a patient with Scleroderma [43]. Our previous observations suggest that normal skin has a very small cellular peak at 50 Hz whereas AK, BCC and SCC have larger peaks at 50 compared to normal skin and new peaks at 80 Hz, 130 Hz and 260 Hz. The results of this study suggest that previous reports [39,40,41,42] indicate that both cells and cancerous tissues are stiffer than normal tissues and reflect changes in the 50 Hz (cellular) and 260 Hz (fibrotic collagen) resonant frequency peaks.

The three most obvious changes that are seen as results of this study are: (1) a new “cellular peak” at a higher resonant frequency (80 Hz) and elastic modulus (1.91 MPa); (2) a new “vascular peak” at 130 Hz with a modulus of 4.3 MPa; and (3) the appearance of a new “fibrous tissue” peak at 260 Hz with a modulus of about 15 MPa. One possible origin of these new peaks is a result of the epithelial–mesenchymal transition (EMT) that was reported to occur in skin cancers [44]. The possible origins of each of these new peaks can be hypothesized based on the cancer literature [39,40,41,42,43,44,45,46,47,48,49,50,51,52,53,54,55,56].

### 4.1. The 80 Hz Peak

The principal cell–cell connections that sense and respond to tensile forces intercellularly as a result of tension applied by the surrounding extracellular matrix are adherens junctions (AJs) [45,46,47,48]. It was proposed that increased cell stiffness associated with cancerous tumors is a result of the stiffening of the AJs between cells of the lesion [48]. AJs are cell–cell adhesions formed by interactions between the extracellular domains of classical cadherins from neighboring cells. Cadherins form trans-dimers by binding to cadherins on adjacent cells via their extracellular domains [47]. Intercellular binding of cadherin molecules stabilize intercellular junctions and provide stress transfer between the collagen fibers in the ECM and the cell cytoskeleton [48]. Therefore, it is possible that changes associated with AJs in a tumor mass give rise to increased stiffness and an increase in the resident frequency of tumor cells.

There are 100 different types of cadherins in vertebrates; they include major classical and non-classical subtypes. E- and N-cadherins are classical subtypes that are transmembrane components of the AJs [47]. E-cadherin is expressed broadly in epithelia. It is often lost during carcinoma progression. N-cadherin is preferentially expressed in non-epithelial cells [44]. Mesenchymal cells express various cadherins, including N-cadherin, R-cadherin and cadherin-11 which are involved in cell migration [49,50]. The conversion of E-cadherin to N-cadherin has been associated with the ability of epithelial cells to form cancerous tumors that metastasize [49]. The neoplastic cellular transformation includes changes in cell–cell interactions, limited proliferation after the establishment of cell–cell contacts, and promotion of cell motility. It is suggested that rearrangements of AJs may change the adhesive function of E-cadherin and play an active role in the migratory activity of carcinoma cells [44,49]. N-cadherin is expressed in highly invasive tumor cell lines that lack E-cadherin expression. The increase in metalloproteinase production by N-cadherin–expressing cells may endow them with a greater ability to penetrate matrix protein barriers [49].

E-and N-cadherins are markers of epithelial–mesenchymal transition (EMT), associated with tumor progression and metastasis [44]. During EMT, epithelial cells lose cell polarity and cell adhesiveness and gain some migratory and invasive properties to become mesenchymal cells [44]. While most of the published work continues to focus on the switch of E- to N-cadherin and its role in the epithelial–mesenchymal transition, other subsets of cadherins (cadherin 17, cadherin 5 and cadherin 6) that have an RGD (arginine–glycine–aspartic acid) binding site in the extracellular domains may play a role in tumor formation [50]. The results reported in this study suggest that EMT may be associated with changes in the cellular ECM; these changes can be quantitatively analyzed by measuring increases in the biomechanical properties of the ECM.

It is postulated that the switch from epithelial-like AJs to mesenchymal-like AJs initiate differences in cell-to-cell adhesion leading to stiffer, tighter cell-to-cell attachments in tumors that would explain the increased cell stiffness of cancer cells. This transformation may also ultimately promote the production and release of metalloproteinases that result in inflammation giving rise to new small blood vessels and the new peak at 130 Hz.

### 4.2. The 130 Hz Peak

Normal blood vessels and veins both have a resonant frequency peak of about 150 Hz and are tethered together to prevent stress concentration at vascular interfaces [51]. Angiogenesis associated with tumors usually arises from capillaries or small blood vessels rather than larger vessels. Tumor blood vessels are more heterogeneous in nature, are tortuous, circumferentially enlarged, irregular, and hyperpermeable [52]. During tumor angiogenesis, pericyte detachment also contributes to the leaky, unstable phenotype of tumor blood vessels [52]. These unstable new blood vessels are likely to have a lower stiffness and be the structural component with a resonant frequency of 130 Hz.

Hippo and two of its main effectors, Yes-associated protein (YAP) and its paralog transcription activator with a binding motif (TAZ), were shown to play critical roles during angiogenesis [53]. It was proposed that YAP/TAZ play major roles in tumor vascular mimicry (VM) which is the formation of blood supply by tumor cells rather than by endothelial cells [53]. Cancer-associated mesenchymal stem cells (MSCs) have higher YAP expression. When the YAP gene is removed from MSCs, proliferation, migration, invasion, and pro-angiogenic ability are inhibited. VM was shown in a variety of cancers including melanoma, breast and lung cancer, ovarian cancer, osteosarcoma, gastric cancer, bladder cancer, hepatocellular cancer, and colorectal cancer [53].

Therefore, it is hypothesized that the new peak at 130 Hz is a result of the formation of new blood vessels by the tumor. This new angiogenesis is largely independent of the existing vascular tissue which is reflected by the presence of both 130 and 150 Hz vascular peaks in BCC and SCC. In addition, the new vascular peak is less stiff than the normal vessels, supporting the observation that the tumor-associated blood vessels are less stable.

### 4.3. The 260 Hz Peak

**Figure 4:**
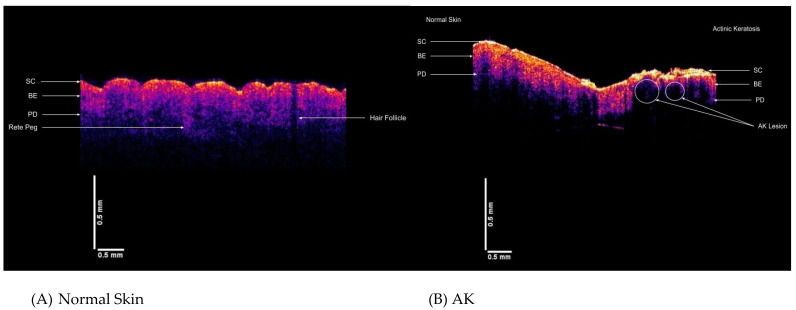

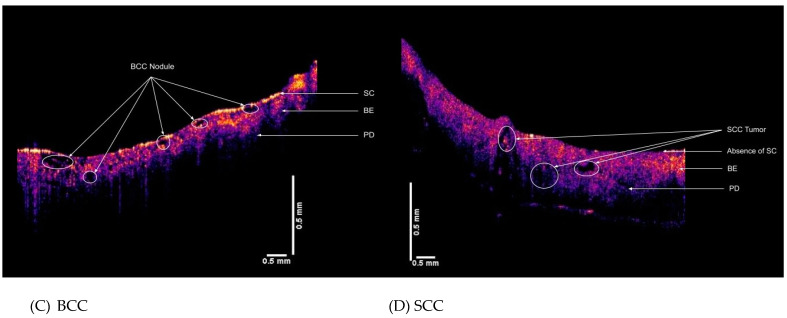
Comparison of color-coded OCT images for (**A**) normal skin, (**B**) AK, (**C**) BCC and (**D**) SCC lesions. In normal skin (**A**) the stratum corneum (SC), basal epithelium (BE) and papillary dermis are (PD) are characteristic of the skin along with the undulating rete pegs. In the AK lesion (**B**) normal skin is seen on the left while the pre-cancerous lesion is seen on the right. In the region of the AK lesion on the right, the basal epithelium and papillary dermis are truncated and replaced with a tumor containing fibrous tissue that reflects light. However, the resonant frequency of the fibrous collagen deposited and the peak height are different than what is seen in cancerous lesions (see Figure 3). The stratum corneum (SC) is thickened and is hypercellular. In BCC (**C**) and SCC (**D**) the stratum corneum (SC) is reduced in size or fragmented and the lesion is seen as either circular or elongated nodules. The rete pegs are missing and black lesions are embedded in the epidermis. There are some basal epithelium (BE) and the papillary dermis (PD) that are reduced in size in the lesion on the right.

During tumor remodeling, the tumor generates a fibrous tissue with many new stromal proteins [41] which can further promote cancer progression [54]. One of the major proteins found in tumor fibrous tissue is fibroblast synthesized collagens [55], High expression of collagens is associated with tumor metastasis in breast cancer [54,55,56] and women with collagen-rich dense breasts have an increased risk of developing breast cancer [54]. Stromal cells can induce increased endothelial cell proliferation and microvessel density [55]. This increased deposition of fibrous collagen and the fibrous collagen resonant frequency peak at 260 Hz would lead to increased fibrous collagen stiffness observed for BCC and SCC in Figure 4. Why cancer cells deposit fibrous stiff collagen (resonant frequency of 260 Hz) as opposed to dermal collagen (resonant frequency of 100 Hz) is unclear but this also occurs during wound healing and genetic diseases of the ECM [43,56,57].

Therefore, it is postulated that the 260 Hz resonant frequency peak is a reflection of the fibrous tissue being deposited in cancerous tumors as a result of the E to M transition and tumor-derived angiogenesis. We recently reported that fibrous tissue in healing wounds [57] and in the skin of a Scleroderma patient [43] have resonant frequencies of between 200 and 220 Hz. It appears that the resonant frequency and stiffness of tumor-associated fibrosis may be higher than that of wound tissue and may reflect the compaction of the tumor as a result of the high cellular and vascular content.

## 5. Conclusions

Our results suggest that biomechanical properties of cellular, vascular and fibrotic tissue in skin lesions are quite different than those in normal skin and may be a means for non-invasively evaluating the quality of the ECM. The cellular resonant frequency peak (and stiffness) is increased from 50 Hz in normal skin to about 80 Hz, in pre- and cancerous lesions. The peaks at 130 Hz and 260 Hz are associated with the deposition of varying amounts of new blood vessels and fibrous tissue in BCC and SCC lesions. Based on the results of this study, VOCT can be used to non-invasively differentiate between benign and cancerous skin lesions based on the appearance of new cellular, vascular, and fibrous resonant frequency peaks and changes in the biomechanical properties of the stroma that surrounds skin epithelial cells.

## Figures and Tables

**Table 1:** Statistical analysis for *p*-values for VOCT measurement for weighted displacement peak heights in micrometers for audible sound frequency of 80 Hz *.

Parameter	Normal Skin	AK	BCC	SCC
Sample Size	59	7	55	46
Average (SD)	4.48 × 10^4^ (4.73 × 10^4^)	1.431 × 10^5^ (1.179 × 10^5^)	9.08 × 10^4^ (9.73 × 10^4^)	9.10 × 10^4^ (9.79 × 10^4^)
Normal Skin	NA	**0.035**	**7.74 × 10^−3^**	**0.01**
AK		NA	0.148	0.15
BCC			NA	0.49

**Values in bold are statistically significant**. * *t*-Test performed with one-tailed distribution and two-sample unequal variances. NA = not applicable.

**Table 2:** Statistical analysis for *p*-values for VOCT measurement for weighted displacement peak heights in m for audible sound frequency of 260 Hz *. The sample size is the same as in Table 1 for each group.

	Normal Skin	AK	BCC	SCC
Average (SD)	9.48 × 10^4^ (1.920 × 10^5^)	3.60 × 10^3^ (9.54 × 10^3^)	1.283 × 10^5^ (1.407 × 10^5^)	7.88 × 10^4^ (1.009 × 10^5^)
Normal Skin	NA	**0.049**	0.274	0.38
AK		NA	**1.24 × 10^−8^**	**5.22 × 10^−6^**
BCC			NA	**0.021**

**Values in bold are statistically significant**. * *t*-Test performed with one-tailed distribution and two-sample unequal variances. NA = not applicable.

**Table 3:** Statistical analysis for *p*-values for VOCT measurement for ratios for weighted displacement peak heights in m for audible sound frequency of 80 Hz/100 Hz *. The sample size is the same as in Table 1 for each group.

	Normal Skin	AK	BCC	SCC
Average (SD)	0.30 (0.28)	0.88 (0.56)	1.160 (0.89)	1.064 (0.77)
Normal Skin	NA	**0.022**	**1.62 × 10^−8^**	**1.36 × 10^−7^**
AK		NA	0.132	0.21
BCC			NA	0.28

**Values in bold are statistically significant**. * *t*-Test performed with one-tailed distribution and two-sample unequal variances. NA = not applicable.

**Table 4:** Statistical analysis for *p*-values for VOCT measurement for ratios of weighted displacement peak heights in m for audible sound frequency of 130 Hz/100 Hz *. The sample size is the same as in Table 1 for each group.

Normal Skin	AK	BCC	SCC
0.30 (0.38)	0.81 (0.54)	3.38 (0.69)	1.186 (0.85)
NA	**0.02**	**1.04 × 10^−6^**	**8.82 × 10^−7^**
	NA	0.13	0.07
		NA	0.26

**Values in bold are statistically significant**. * *t*-Test performed with one-tailed distribution and two-sample unequal variances. NA = not applicable.

**Table 5:** Statistical analysis for *p*-values for VOCT measurement for weighted displacement peak heights in m for audible sound frequency of 260 Hz/100 Hz *. The sample size is the same as in Table 1 for each group.

	Normal Skin	AK	BCC	SCC
Average (SD)	0.36 (0.36)	0.122 (0.33)	2.27 (4.55)	1.574 (1.401)
Normal Skin	NA	0.057	**1.89 × 10^−3^**	**1.50 × 10^−6^**
AK		NA	**5.70 × 10^−4^**	**1.60 × 10^−7^**
BCC			NA	0.144

**Values in bold are statistically significant**. * *t*-Test performed with one-tailed distribution and two-sample unequal variances. NA = not applicable.

**Table 6:** Statistical analysis for *p*-values for VOCT measurement for ratios of weighted displacement peak heights in m for audible sound frequency of 150 Hz/100 Hz *. The sample size is the same as in Table 1 for each group. NA = not applicable.

	Normal Skin	AK	BCC	SCC
Average (SD)	0.89 (1.094)	0.49 (0.23)	0.88 (0.65)	0.88 (0.56)
Normal Skin	NA	0.08	0.43	0.43
AK		NA	**0.004**	**0.004**
BCC			NA	0.49

**Values in bold are statistically significant**. * *t*-Test performed with one-tailed distribution and two-sample unequal variances. NA = not applicable.

## Data Availability

The data supporting the results can be found at optovibronex.com.

## Abbreviations

OCT	optical coherence tomography
VOCT	vibrational optical coherence tomography
H&E	hematoxylin and eosin
ECM	extracellular matrix
AK	actinic keratosis
BCC	basal cell carcinoma
SCC	squamous cell carcinoma
VM	vascular mimicry
YAP	yes-associated protein
TAZ	transcription activator with PDZ binding motif
EMT	epithelial–mesenchymal transition
